# Influenza Vaccine Effectiveness in Mainland China: A Systematic Review and Meta-Analysis

**DOI:** 10.3390/vaccines9020079

**Published:** 2021-01-23

**Authors:** Xiaokun Yang, Hongting Zhao, Zhili Li, Aiqin Zhu, Minrui Ren, Mengjie Geng, Yu Li, Ying Qin, Luzhao Feng, Zhibin Peng, Zhijie An, Jiandong Zheng, Zhongjie Li, Zijian Feng

**Affiliations:** 1 Division of Infectious Disease, Key Laboratory of Surveillance and Early-Warning on Infectious Disease, Chinese Center for Disease Control and Prevention, Beijing 102206, China; yangxk@chinacdc.cn (X.Y.); zhaoht@chinacdc.cn (H.Z.); lizl@chinacdc.cn (Z.L.); renmr@chinacdc.cn (M.R.); gengmj@chinacdc.cn (M.G.); liyu1@chinacdc.cn (Y.L.); Qinying@chinacdc.cn (Y.Q.); pengzb@chinacdc.cn (Z.P.); 2 Division of Infectious Disease Prevention and Disinfection Management, Shanghai Pudong New Area Center for Disease Control and Prevention, Shanghai 200136, China; zhuaiqin28@163.com; 3 School of Population Medicine and Public Health, Chinese Academy of Medical Sciences and Peking Union Medical College, Beijing 100730, China; fengluzhao@cams.cn; 4 National Immunization Program, Chinese Center for Disease Control and Prevention, Beijing 102206, China; anzjl@chinacdc.cn; 5 Chinese Center for Disease Control and Prevention, Beijing 102206, China

**Keywords:** influenza, vaccine effectiveness, China, systematic review, meta-analysis

## Abstract

Influenza endangers human health but can be prevented in part by vaccination. Assessing influenza vaccine effectiveness (VE) provides scientific evidence for developing influenza vaccination policy. We conducted a systematic review and meta-analysis of studies that evaluated influenza VE in mainland China. We searched six relevant databases as of 30 August 2019 to identify studies and used Review Manager 5.3 software to analyze the included studies. The Newcastle–Ottawa scale was used to assess the risk of publication bias. We identified 1408 publications, and after removing duplicates and screening full texts, we included 21 studies in the analyses. Studies were conducted in Beijing, Guangzhou, Suzhou, and Zhejiang province from the 2010/11 influenza season through the 2017/18 influenza season. Overall influenza VE for laboratory confirmed influenza was 36% (95% CI: 25–46%). In the subgroup analysis, VE was 45% (95% CI: 18–64%) for children 6–35 months who received one dose of influenza vaccine, and 57% (95% CI: 50–64%) who received two doses. VE was 47% (95% CI: 39–54%) for children 6 months to 8 years, and 18% (95% CI: 0–33%) for adults ≥60 years. For inpatients, VE was 21% (95% CI: −11–44%). We conclude that influenza vaccines that were used in mainland China had a moderate effectiveness, with VE being higher among children than the elderly. Influenza VE should be continuously monitored in mainland China to provide evidence for policy making and improving uptake of the influenza vaccine.

## 1. Introduction

Influenza is an acute respiratory infectious disease that causes a large burden of disease globally. According to WHO estimates, 5 to 10% of adults and 20 to 30% of children will suffer from seasonal influenza infection every year, resulting in 3 to 5 million cases of severe illness and 290,000–650,000 deaths [1]. In each year of the 2010/2011 through 2014/2015 seasons, there was an estimated 65 to 190 million people infected and 88,100 influenza-associated excess respiratory deaths in China [2]. Although there are antiviral drugs for influenza, such as oseltamivir and zanamivir [3], influenza vaccination is considered the most economical and effective way to prevent influenza [4]. However, with the exception of a few cities where local government subsidize influenza vaccination programs, influenza vaccination has not been introduced in a national, government-funded program for people in mainland China, nor is the influenza vaccine included in health insurance. People pay out-of-pocket for the influenza vaccine, which may contribute to China’s extremely low vaccine uptake rate of 1.5 to 2.2% [5]. Health and economic analyses of seasonal influenza vaccination may help local and national governments justify influenza vaccination programs. An essential component of the health and economic evaluation of vaccines is their effectiveness. Influenza vaccine effectiveness varies year to year. Monitoring influenza VE can provide evidence for program and policy making.

Methods for determining influenza VE vary significantly. Some studies have evaluated the safety and immunogenicity of the influenza vaccine [6,7,8,9] and some influenza VE studies based on influenza-like illness (ILI), which may underestimate influenza VE [10,11,12]. The use of laboratory diagnosed influenza for VE studies has been facilitated by widespread adoption of rapid laboratory testing for influenza virus in medical institutions at all levels of China [13,14,15,16]. We report the results of a systematic review and meta-analysis of influenza VE studies in mainland China to provide evidence for improving VE monitoring and support influenza vaccine policy making.

## 2. Materials and Methods

### 2.1. Literature Retrieval

We used the key words “influenza,” “influenza vaccination”, “vaccine effectiveness”, and “China” as search terms to locate published articles. We searched the Wanfang Database, China National Knowledge Infrastructure (CNKI), China Biology Medicine (CBM), and VIP journal database for Chinese language studies; we searched PubMed and Web of Science for English language studies. We used literature reference tracing to identify additional articles and reduce the chance of omitting studies meeting the eligibility criteria.

### 2.2. Eligibility Criteria

To be eligible for this systematic review and meta-analysis a study had to meet the following criteria: (1) the study setting was in mainland China; (2) the study design was either a test-negative design (TND) case–control study, another type of case–control study, or a cohort study; (3) patients with ILI or acute respiratory infection (ARI) had to have been tested by reverse transcription polymerase chain reaction(RT-PCR) for the influenza virus; (4) the study reported influenza VE; and (5) the study was a post-marketing influenza VE evaluation.

### 2.3. Data Retrieval

We developed a spreadsheet to organize extracted data. Authors X.Y. and H.Z. extracted data from the included studies; Z.L., M.R., and M.G. checked the data for completeness and accuracy; reviewer disagreements were resolved by Z.L. Information extracted included title, first author, year of publication, study period, study site, study design, study participant description, subject inclusion criteria, samples obtained, and vaccination rate of the influenza positive group and the influenza negative group.

### 2.4. Literature Quality Evaluation

We used the Newcastle–Ottawa Scale (NOS) [17] to evaluate the quality of the eligible studies from three aspects—population selection, comparability, and exposure evaluation—each aspect receiving up to three points for a total possible score of nine points. We considered scores of more than six points to indicate studies of high quality with low risk of bias; scores of four or five points indicated moderate quality with average risk of bias; scores of three points or fewer indicated low quality with high risk of bias.

### 2.5. Statistical Analyses

We used Review Manager 5.3 software to conduct meta-analyses. Heterogeneity was assessed by the *I*^2^ statistic. The value of *I*^2^ reflects the percent of heterogeneity in the total variation; an *I*^2^ greater than 50% is evidence of heterogeneity. In our testing for heterogeneity, we used a fixed-effect model (*p* > 0.05 and *I*^2^ < 50%) or a random-effect model (*p* ≤ 0.05 or *I*^2^ ≥ 50%) [18]; we present results as odds ratios (*OR*) with 95% confidence intervals (CI) and display results in forest plots. We conducted subgroup analyses with delimited data. We compared changes in I^2^ statistic values when studies with the highest VE, the lowest VE, and the largest sample size were excluded. We considered the results to be stable if the *I*^2^ statistic change was <10%. Influenza VE was calculated as (1 − odds ratio) ×100%, where the odds ratio is the odds of vaccination in cases divided by the odds of vaccination in controls.

## 3. Results

### 3.1. Basic Information of Eligible Studies

**Figure 1:**
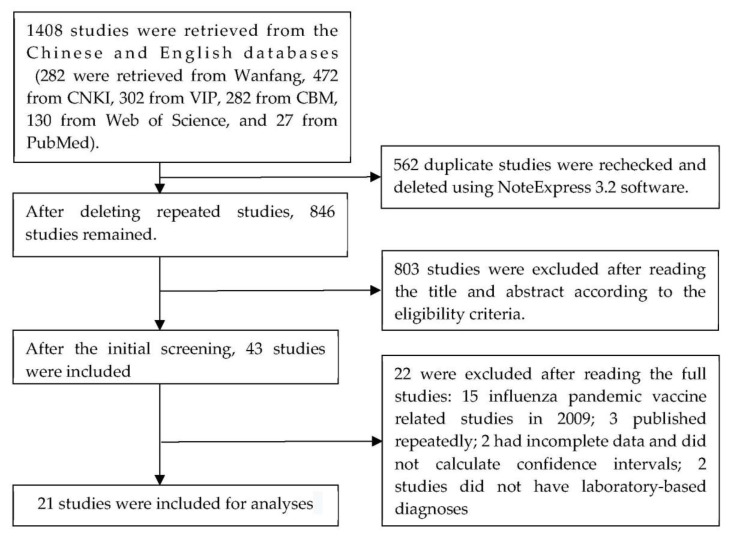
Literature screening process for systematic reviews and meta-analysis.

The search terms identified 1408 studies from the six databases—282 from the Wanfang database, 472 from CNKI, 302 from VIP journal database, 282 from CBM, 130 from Web of Science, and 27 from PubMed. Our eligibility review identified 21 articles for including in the analyses [19,20,21,22,23,24,25,26,27,28,29,30,31,32,33,34,35,36,37,38,39] (Figure 1). The 21 eligible studies were conducted between the 2010–2011 influenza season and the 2017–2018 influenza season. The most common settings were: Beijing; Guangzhou, Guangdong province; Suzhou, Jiangsu province; and Yongkang and Yiwu in Zhejiang province. Study subjects in the Guangzhou studies were children aged 6 months to 8 years old; subjects in Beijing studies included outpatients and hospitalized patients of all ages; subjects in Suzhou studies were 3-to-6-year-old preschool children. Research methods included case–control studies and TND studies. Descriptions of the eligible studies are shown in Table 1.

### 3.2. Quality Evaluations

Based on Newcastle–Ottawa Scale quality evaluation of the 21 studies (including 26 research results), ten studies received seven points, two studies received six points, and 14 studies received five points. The two most common reasons for score deductions were representativeness of the influenza positive group and absence of a reported response rate. Scores are shown in Table 2.

### 3.3. Meta Analysis of Influenza VE

**Figure 2:**
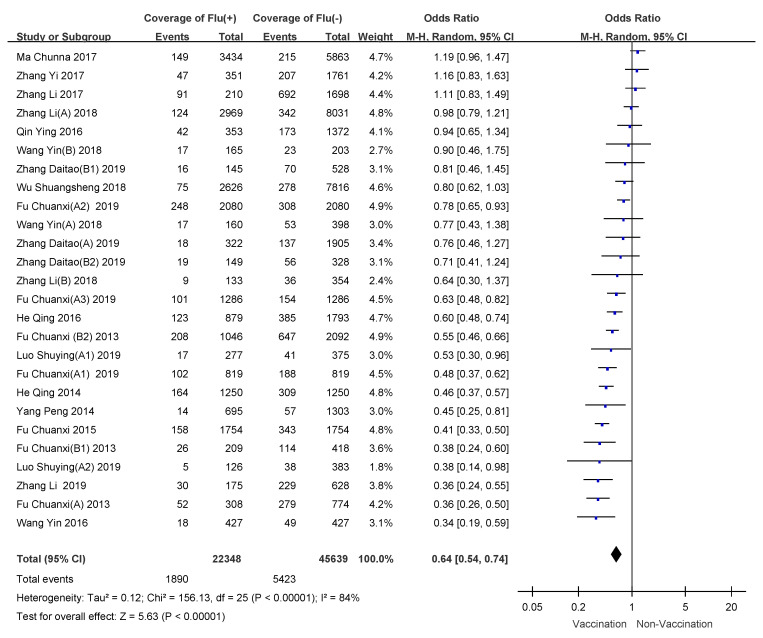
Meta-analysis of influenza vaccine effectiveness (VE) in mainland China.

Meta-analysis was performed on the 21 included studies (26 separate research results). The heterogeneity test showed that *I*^2^ = 84%, *p* < 0.05, therefore, we used a random-effects model for merging data. Review Manager 5.3 determination showed the overall OR to be 0.64 (95% CI: 0.55–0.75), for a VE of 36% (95% CI: 25–45%). See Figure 2.

### 3.4. Subgroup Analyses

**Figure 3:**
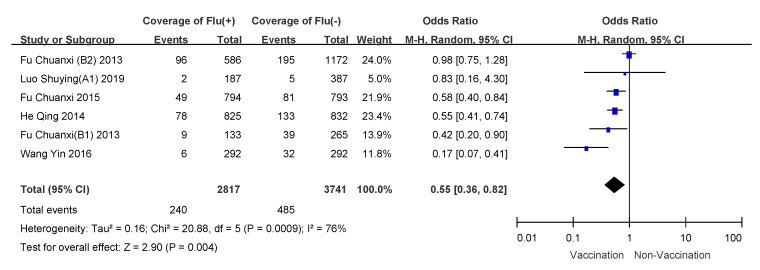
Subgroup analysis of children 6–35 months who received one dose of influenza vaccine.

**Figure 4:**
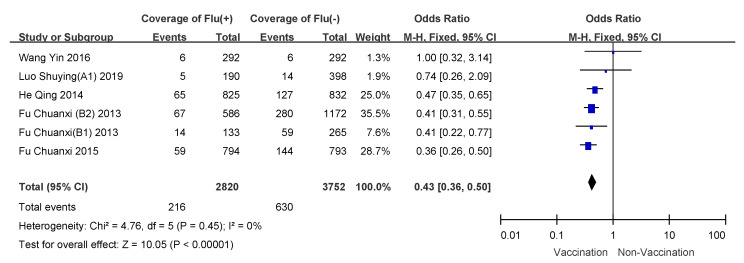
Subgroup analysis of children 6–35 months who received two doses of influenza vaccine.

**Figure 5:**
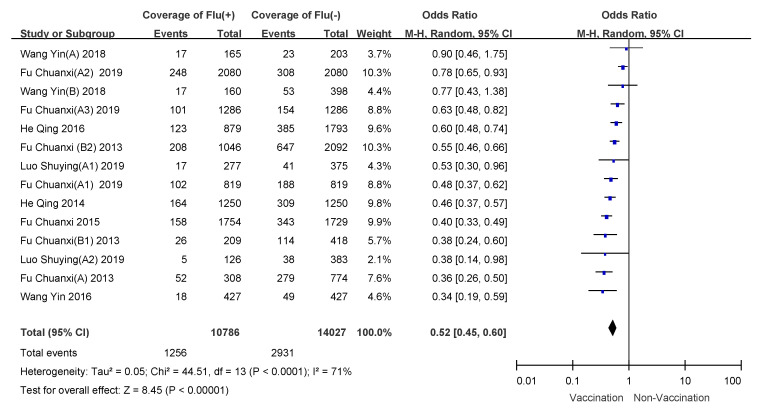
Subgroup analysis of children aged 6 months to 8 years old.

**Figure 6:**
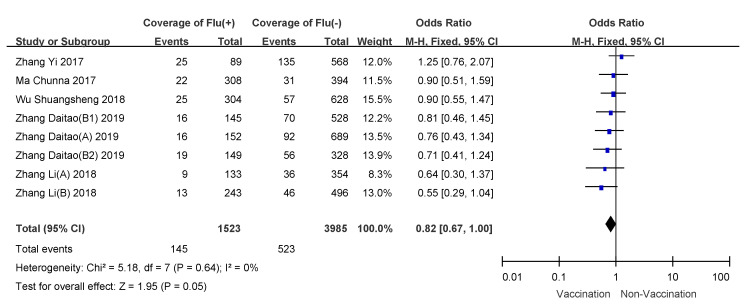
Subgroup analysis of adults more than 60 years old.

**Figure 7:**
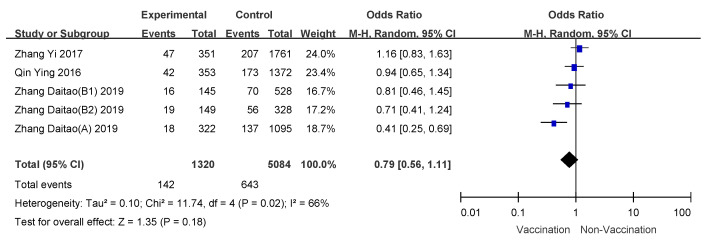
Subgroup analysis of inpatients.

**Figure 8:**
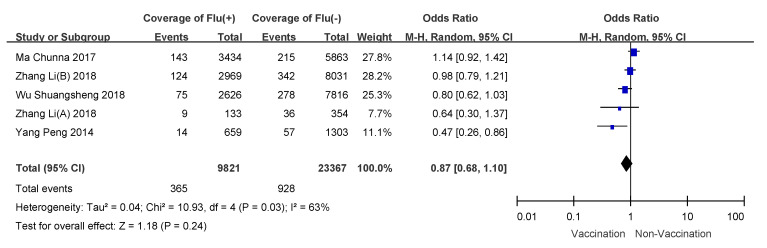
Subgroup analysis of outpatients.

We conducted subgroup analyses using age of vaccinee, number of doses, and disease severity (hospitalized patients or outpatients). VE for 6 month to 35 month old children who received two doses of influenza vaccine was 57% (95% CI: 50–64%); overall VE for children 6 months to 8 years of age was 47% (95% CI: 39–54%); VE for people over 60 years old was the lowest, and VE for hospitalized patients was 21% (95% CI: −11–44%). Detailed results are shown in Table 3, and meta-analysis results are shown in Figure 3, Figure 4, Figure 5, Figure 6, Figure 7 and Figure 8.

### 3.5. Sensitivity Analysis and Publication Bias

**Figure 9:**
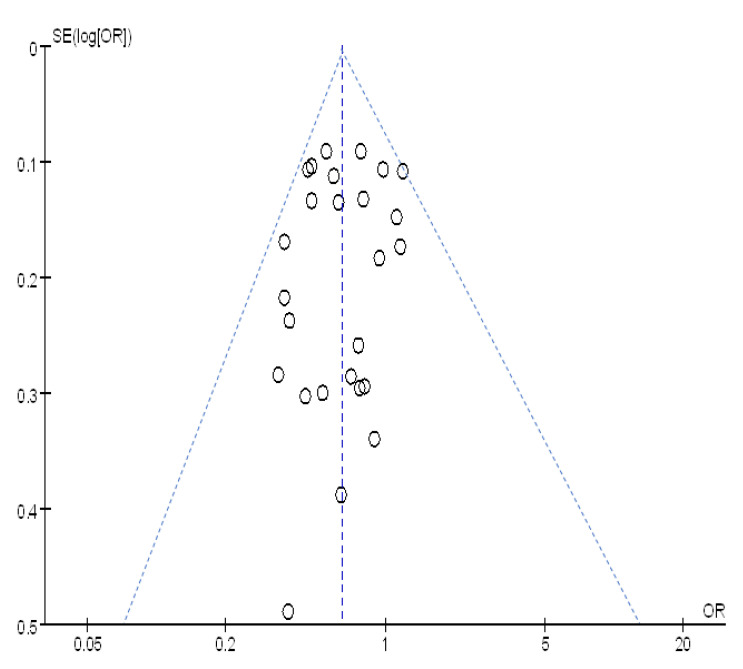
Funnel plot for meta-analysis of influenza VE.

The highest estimated influenza VE reported in the included studies was 66% (95% CI: 41–81%) by Wang Yin and colleagues [37]. We excluded this study and re-conducted the meta-analysis, finding an OR of 0.65 (95% CI: 0.56–0.76), for a VE of 35% (95% CI: 23–44%). Ma Chunna and colleagues reported the lowest influenza VE [29], which was −19% (95% CI: −47–4%), and had the study with the largest sample size. After excluding Ma’s study, the recalculated OR was 0.62 (95% CI: 0.54–0.72), for a VE of 38% (95% CI: 28–46%). The difference in ORs before and after excluding the studies was less than 10%, implying stability of the meta-analyses. Figure 9 shows a scatter plot (funnel plot) that plots OR on the horizontal axis and the standard error of log(OR) on the vertical axis. The distribution of scattered points was approximately symmetric, indicating that risk of publication bias was relatively low.

## 4. Discussion

Influenza vaccination is widely considered to be the most effective and cost-beneficial way to prevent influenza [4]. Evaluating influenza VE is very important for supporting policy making. We conducted a meta-analysis of 21 published studies set in mainland China and found that the overall VE was 36% during the 2010/2011 through 2017/2018 influenza seasons, showing a moderately protective effect of vaccination. Influenza VE was higher (57%) among young children 6 to 35 months of age who received two doses influenza vaccine for their first time receiving influenza vaccine; VE was 45% among same-age children who received one dose of influenza vaccine. We found that influenza VE was higher among children than among individuals over 60 years of age.

Two studies from the United States found that during the 2003–2005 season and the 2005–2007 season, VE for 6 to 59 month children who received two doses of vaccine was 57% (95% CI: 28–74%) and 56% (95% CI: 25–74%) [40,41], which is consistent with our findings. However, among children in the two U.S. studies who received a single dose, the vaccine showed no significant protective effect. This is in contrast to our meta-analysis that included six studies and found a protective effect with one dose—although lower than for two doses. This may be due in part to a larger sample size in our meta-analysis that could provide more statistical power.

A retrospective study in Italy [42] found that influenza VE in children was 37.1% (95% CI: 22.2–49.2%) during the 2010/2011 to 2017/2018 influenza seasons. Our findings of higher VE may be because we did not adjust for age, gender, and epidemic season in our meta-analyses, while the study conducted in Italy did. Both studies showed better protection for children from 2010/2011 through 2017/2018 seasons than for elderly adults. Using a fixed-effect model, we found that for people over 60 years of age, VE was only 18%. Such a low protective effect is unsatisfactory. Lower VE among the elderly may be due to immunosenescence. Studies have shown that vaccine-mediated antibody titers among the elderly are lower than those among younger individuals [43,44,45,46]. Different influenza vaccination strategies for the elderly are needed to overcome immunosenescence. The US Food and Drug Administration (FDA) approved a high-dose trivalent inactivated influenza vaccine in 2009, increasing each antigen component from the standard dose of 15 μg to 60 μg [47]. High-dose influenza vaccines demonstrate better efficacy against laboratory-confirmed influenza [48,49,50,51] and better effectiveness against confirmed influenza, influenza-related medical visits, hospitalization, and death [52,53,54]. The findings from our study support the need for promoting development and introduction of high-dose influenza vaccines and adjuvant vaccines that are more suitable for the elderly in mainland China.

A 2016 review found that influenza VE, assessed with test-negative design studies in inpatient settings, had similar results to TND assessments in outpatient settings [55]. A study set in Spain found that influenza VE was 34% (95% CI: 6–54%) among outpatients and 32% (95% CI: 15–45%) among hospitalized patients during the 2010/2011 to 2015/2016 seasons [56]. A study from the U.S. among adults over 18 years of age conducted from 2015 to 2018 [57] showed that influenza VE was 31% (95% CI: 26–37%) for outpatients and 36% (95% CI: 27–44%) for inpatients. We used a random-effect model to evaluate VE for outpatients and inpatients and found that VE was 21% (95% CI: −11–44%) for inpatients and 13% (95% CI: −10–32%) for outpatients—lower than the findings from these other studies. It is possible that vaccine-circulating-strain mismatch is partially responsible. For example, for the 2014/2015 influenza season, the match was poor, and Ma Chunna and colleagues [29] found that influenza vaccination had no protective effect among outpatients. Zhang Yi and colleagues [30] also found no protective effect among hospitalized patients of all age groups in Beijing in the 2015/2016 influenza season. The reason for the poor VE, could also be related to the extremely low influenza vaccination rate in mainland China. Low coverage makes VE assessment more difficult, since VE study sample sizes need to be larger when vaccination rates are low. Evaluation of influenza VE will be facilitated by conducting studies in cities with higher vaccination coverage levels, which currently tend to be cities in which local governments sponsor seasonal influenza vaccination among targeted groups, such as school children or the elderly.

Our study has several limitations. First, the studies we included made some statistical adjustments in their OR calculations, but the adjusting factors were not the same in all studies. Therefore, we did not use adjustment factors in Revman 5.3 software for our meta-analysis. Second, our study only involved only two age groups—6 months to 8 years and over 60 years—limiting our ability to conduct additional subgroup analyses. Third, we did not analyze VE by influenza virus types, primarily due to lack of information about virus types in the studies. Using a standardized protocol in the future, we should be able to assess the VE of different ages and different virus types. Fourth, too few studies were based on laboratory confirmed cases of influenza in mainland China—only Beijing, Suzhou, Zhejiang and Guangzhou regularly used laboratory confirmation. The settings for our meta-analysis were in northern and southern China, which misses some variation in the epidemic seasons in mainland China. Establishing a wider area for VE studies will be useful in the future. Finally, we did not analyze VE by influenza season due to an insufficient number of VE studies.

There are many challenges and opportunities for comprehensive evaluation of influenza VE in mainland China [58]. Vaccine-circulating-strain mismatch, vaccine type and technology, virus strains, vaccination season, repeated vaccination, northern vs. southern influenza seasons, among other factors can be used to understand the many influences of influenza VE. It is essential to evaluate influenza VE across a much larger scale and with systematic and strategic use of a unified research protocol.

## 5. Conclusions

Based on published studies set in mainland China that used laboratory-confirmed influenza to evaluate influenza VE, we found that influenza vaccination has a moderate protective effect against medically-attended influenza, and that influenza VE among children is higher than among the elderly. Influenza VE evaluations should be conducted continuously, and with a unified research protocol to provide a scientific basis for formulation and adjustment evidence-based influenza vaccination policy.

## Figures and Tables

**Table 1:** Characteristics of 21 studies included in the meta-analysis.

(Ref.)	Published Year	Study Period	Study Place	Study Objects	Study Method	Vaccination Rate in Case Group	Vaccination Rate in Control Group
[19]	2013	2010/09–2011/09	Guang Zhou	6 months~5 years community children	case–control study	16.9% (52/308)	36.0% (279/774)
[20]	2013	2011/01–2011/06	Guang Zhou	6 months~5 years community children	1:2 case–control study	12.4% (26/209)	27.3% (114/418)
[20]	2013	2012/01–2012/06	Guang Zhou	6 months~5 years community children	1:2 case–control study	19.9% (208/1046)	30.9% (647/2092)
[21]	2014	2013/02–2013/06	Guang Zhou	6 months~5 years community children	1:1 case–control study	13.1% (164/1250)	24.7% (309/1250)
[22]	2015	2013/02–2013/06	Guang Zhou	8 months~6 years community children	1:1 case–control study	9.0% (158/1754)	19.6% (343/1754)
[23]	2016	2014/02–2014/07	Guang Zhou	6 months~5 years community children	1:2 case–control study	14.0% (123/879)	21.5% (385/1793)
[24]	2019	2014/02–2014/07	Guang Zhou	6 months~8 years community children	1:1 case–control study	12.5% (102/819)	23.0% (188/819)
[24]	2019	2015/03–2015/07	Guang Zhou	6 months~8 years community children	1:1 case–control study	12.0% (248/2080)	14.8% (308/2080)
[24]	2019	2016/03–2016/05	Guang Zhou	6 months~8 years community children	1:1 case–control study	7.9% (101/1286)	12.0% (154/1286)
[25] *	2014	2012/12–2013/01	Bei Jing	Outpatients of all age groups	Test-negative design	2.0% (14/695)	4.4% (57/1303)
[26]	2018	2013/11–2014/04	Bei Jing	Outpatients of more than 60 years old	Test-negative design	6.8% (9/133)	10.2% (36/354)
[27]	2016	2013/12–2015/05	Bei Jing	Inpatients of all age groups	Test-negative design	11.9% (42/353)	12.6% (173/1372)
[28]	2017	2014/11–2014/12	Bei Jing	6~18 years old students	case–control study	43.3% (91/210)	40.8% (692/1698)
[29]	2017	2014/11–2015/04	Bei Jing	Outpatients of all age groups	Test-negative design	4.3% (149/3434)	3.7% (215/5863)
[30] *	2017	2015/10–2016/05	Bei Jing	Outpatients of all age groups	Test-negative design	13.4% (47/351)	11.8% (207/1761)
[31]	2018	2015/11–2016/03	Bei Jing	Outpatients of all age groups	Test-negative design	4.2% (124/2969)	4.3% (342/8031)
[32]	2018	2016/11–2017/04	Bei Jing	Outpatients of all age groups	Test-negative design	2.9% (75/2626)	3.6% (278/7816)
[33]	2019	2016/11–2017/04	Bei Jing	Outpatients of all age groups	Test-negative design	5.6% (18/322)	7.2% (137/1905)
[34]	2019	2016/11–2017/04	Bei Jing	6~18 years old students	case–control study	17.0% (30/175)	36.5% (229/628)
[35]	2019	2016/11–2017/04	Bei Jing	Inpatients of more than 60 years old	Test-negative design	11.0% (16/145)	13.3% (70/528)
[35]	2019	2017/11–2018/04	Bei Jing	Inpatients of more than 60 years old	Test-negative design	12.8% (19/149)	17.1% (56/328)
[36]	2019	2016/10–2017/04	Zhe Jiang	Outpatients of 6months~6 years	Test-negative design	6.1% (17/277)	10.9% (41/375)
[36]	2019	2017/10–2018/04	Zhe Jiang	Outpatients of 6months~6 years	Test-negative design	4.0% (5/126)	9.9% (38/383)
[37]	2016	2011/10–2012/09	Su Zhou	6 months~5 years community children	1:1 case–control study	4.2% (18/427)	11.5% (49/427)
[38] *	2018	2015/10–2016/02	Su Zhou	Kindergarten children aged 3–6 years	Test-negative design	10.3%(17/165) *	11.3% (23/203) *
[39] *	2018	2016/10–2017/02	Su Zhou	Kindergarten children aged 3–6 years	Test-negative design	10.6%(17/160) *	13.3% (53/398) *

Ref. [25] *: The diagnosis of influenza-positive patients was based on the isolation of influenza viruses from cell cultures; other studies were based on RT-PCR. Ref. [25] * and [30] *: Vaccination information was self-reported; vaccination information of other studies can be found in electronic database of vaccination. Ref. [38] * and [39] *: The original study method was cohort study, but used test-negative design method to analyze, so we showed the positive rate of tested-sample in Table 1.

**Table 2:** Quality evaluation of eligible studies: based on the Newcastle–Ottawa Scale.

[Ref.]	Published Year	Selection				Comparability	Exposure			Total Scores
		Is the Case Definition Adequate?	Representativeness of the Cases	Selection of Controls	Definition of Controls	Comparability of Cases and Controls on the Basis of the Design or Analysis	Ascertainment of Exposure	Same Method of Ascertainment for Cases and Controls	Non-Response Rate	
[19]	2013	1	0	1	1	2	1	1	0	7
[20]	2013	1	0	1	1	2	1	1	0	7
[20]	2013	1	0	1	1	2	1	1	0	7
[21]	2014	1	0	1	1	2	1	1	0	7
[22]	2015	1	0	1	1	2	1	1	0	7
[23]	2016	1	0	1	1	2	1	1	0	7
[24]	2019	1	0	1	1	2	1	1	0	7
[24]	2019	1	0	1	1	2	1	1	0	7
[24]	2019	1	0	1	1	2	1	1	0	7
[25]	2014	1	0	0	1	1	1	1	0	5
[26]	2018	1	0	0	1	1	1	1	0	5
[27]	2016	1	0	0	1	1	1	1	0	5
[28]	2017	1	0	1	0	2	1	1	0	6
[29]	2017	1	0	0	1	1	1	1	0	5
[30]	2017	1	0	0	1	1	1	1	0	5
[31]	2018	1	0	0	1	1	1	1	0	5
[32]	2018	1	0	0	1	1	1	1	0	5
[33]	2019	1	0	0	1	1	1	1	0	5
[34]	2019	1	0	1	1	1	1	1	0	6
[35]	2019	1	0	0	1	1	1	1	0	5
[35]	2019	1	0	0	1	1	1	1	0	5
[36]	2019	1	0	0	1	1	1	1	0	5
[36]	2019	1	0	0	1	1	1	1	0	5
[37]	2016	1	0	1	1	2	1	1	0	7
[38]	2018	1	0	0	1	1	1	1	0	5
[39]	2018	1	0	0	1	1	1	1	0	5

**Table 3:** Influenza VE subgroup analysis.

Study Subjects	Heterogeneity Test: χ^2^ (*P*)	*I* ^2^	Model Selection	OR (95%*CI*)	VE (95%*CI*)
Receipt of Two Doses of Vaccine *					
Children 6–35 months receiving one dose	20.8 (0.0009)	76%	random-effects model	0.55 (0.36–0.82)	45% (18–64%)
Children 6–35 months receiving two doses	4.76 (0.45)	0	fixed-effects model	0.43 (0.36–0.50)	57% (50–64%)
**Age Groups**					
6 months to 8 years	37.4 (0.0003)	65%	random-effects model	0.53 (0.46–0.61)	47% (39–54%)
More than 60 years	5.18 (0.64)	0	fixed-effects model	0.82 (0.67–1.00)	18% (0–33%)
Disease Severity **					
inpatients	11.74 (0.02)	66%	random-effects model	0.79 (0.56–1.11)	21% (−11–44%)
outpatients	10.93 (0.03)	63%	random-effects model	0.87(0.68–1.10)	13% (−10–32%)

* According to the influenza vaccine manufacturer prescribing information, children aged 6–35 months should receive two doses of influenza vaccine. ** Compared to outpatients, inpatients were considered more seriously ill.
